# The Relationship between Internet Use and Population Health: A Cross-Sectional Survey in China

**DOI:** 10.3390/ijerph19031322

**Published:** 2022-01-25

**Authors:** Liqing Li, Haifeng Ding

**Affiliations:** School of Public Administration and Law, Hunan Agricultural University, Changsha 410100, China; liliqing1136@163.com

**Keywords:** Chinese population health, health behavior, Internet use, subjective health, self-rated health, chronic conditions

## Abstract

Given the increasing use of the Internet in China, the relationship between Internet use and population health has been receiving increased academic attention. However, the current literature has not yet reached a unified conclusion; thus, further research is very necessary. This study used the 2018 China Family Panel Studies (CFPS) data to explore the relationship between Internet use and the health of the population and to evaluate the possible heterogeneity of the association between different groups and the mediation path. This study revealed that Internet use has a positive association with the subjective health of the population. The results of the heterogeneity analysis revealed that Internet use is more significantly associated with the health of non-agricultural workers and those with higher education levels than that of agricultural workers and those with lower education levels. Further analysis revealed that health behavior is an important mediator between Internet use and population health. These results remain robust even after using propensity score matching (PSM) to eliminate sample selection bias.

## 1. Introduction

Health is the foundation of a nation and the basis of human life [1,2]. Having benefitted from the dividends of reform and opening up, China’s medical and health situation and the health of the population have been improving, and the average life expectancy is increasing (77.3 years in 2020) [3]. However, with the improvement of living standards and changes in lifestyle, chronic diseases such as hypertension (prevalence 23.2%) and diabetes (prevalence 12.4%), which are known as “wealthy diseases” in China, have greatly affected the health of the Chinese population [4]. According to the latest data from the Chinese Center for Disease Control and Prevention, the number of patients with hypertension in China reached 420 million in 2020, and the number of people who are overweight and obese was as high as 250 million. This is the highest number of overweight and obese people worldwide [5]. To change this situation, the Communist Party of China and the government have attached great importance to this and have introduced a series of policies and measures to reduce the prevalence of chronic diseases in all aspects and to improve the health of the entire population. In 2016, the State Council proposed the “Healthy China 2030 Plan”, which proposed the vigorous promotion of a healthy China and putting people’s health in a strategic position of priority for development [6]. In 2017, President Xi proposed a healthy China strategy at the 19th Congress of the Communist Party of China. This is the first time in Chinese history that health has been raised to the level of national strategy [7]. In 2019, the State Council issued the “Opinions on the Implementation of the Healthy China Action”, which included a systematic strategic plan for the implementation of healthy actions in the future. In 2021, the promotion of a healthy China was included in the outline of China’s 14th Five-Year Plan [8]. The above series of policies shows that improving the national health level will be a topic of focus for China for a long time.

Moreover, the rapid development of Internet technology, represented by the Internet and smartphones, has profoundly changed people’s lives and behaviors [9]. The Internet breaks through traditional time and space restrictions, enhances information exchange, broadens information collection channels, reduces the cost of information acquisition, and provides convenience for life and work [10,11]. Studies have shown that Internet use is closely associated with health behaviors. For example, studies have shown that people who use smartphones are more likely to have college degrees, be more physically active, eat less low-fat diets, and have higher incomes [12,13]. According to the data from the National Bureau of Statistics of China, as of 2020, the number of Chinese Internet users reached 989 million, 986 million of whom are mobile phone users, and the Internet usage rate reached 70.4% [14]. Compared to 2011, the number of Internet users has nearly doubled, and in terms of the absolute number of Internet users, China ranks first in the world. As early as 2015, the State Council issued the “Guiding Opinions on Actively Promoting the ‘Internet + Action’”, which stated the need to vigorously promote the deep integration of the Internet in various fields of society and to make full use of the advantages of the scale and application of the Internet [15].

However, the development of Internet technology remains uneven between urban areas and rural areas because of the dualistic nature of China’s urban–rural distribution [16]. Information infrastructure is weaker in rural areas, and the Internet penetration rate is much lower in rural areas than it is in cities [17]. Agricultural work, which is necessarily time-consuming and physically demanding, and the generally low literacy level in rural areas can limit Internet usage [18]. In addition, people have largely different Internet use behaviors. For example, people with high educational attainment are more likely to use the Internet for health information searches, while those with low educational attainment experience certain barriers [19,20]. Thus, this study focused on the relationship between Internet use and population health to provide new evidence for the further understanding of population health.

This study aimed to (1) explore whether a relationship exists between Internet use and population health; (2) determine the direction of the relationship, if there is any; (3) analyze the differences in Internet use on the health of those engaged in agriculture and those not engaged in agriculture and those with a high school-level of education or less and those with college-level or higher education; and (4) validate the mediating effect of health behavior between Internet use and population health.

## 2. Literature Review

We entered the keywords “Internet use” and “health” to search for relevant papers indexed in the China National Knowledge Infrastructure (CNKI), Web of Science (WOS), PubMed, etc. Finally, 100 relevant papers were obtained. The theory of health ecology states that the health of individuals and populations is the result of the interaction of individual factors and environmental factors [21]. In the context of informatisation, the Internet, as one of the external environmental factors, intervenes through various channels and penetrates all areas of society and has a connection with the health of the population. At present, research on the Internet and population health has been receiving increased academic attention. Specifically, there are mainly three viewpoints.

First, Internet use is positively associated with population health. Bessière et al. found that the growth of the internet has made it more convenient for Americans to obtain medical information and that internet use for communication with friends and family was associated with small but reliable decreases in depression (i.e., increased Internet use for communication with friends and family from 3 to 5 days per week to once a day was associated with a 0.07 standard deviation depression symptoms, *p* = 0.007). [22]. Wang used the 2013 China Comprehensive Social Survey data to find that the Internet has a significant association with the physical and mental health of the older population. The physical and psychological health of older people using the Internet was higher by 31.9% and 18.8%, respectively, compared to older people who did not use the Internet [23]. Yang and Gu found that rural Chinese residents who use the Internet are in better health, and informal support is an important mediating mechanism [24]. Yang and He also showed that Internet use has a positive association with the health of the population [25]. In addition, some studies have focused on the mediating mechanism between Internet use and health. For example, risk perception [26], participation in social activities, reading and self-evaluation of socioeconomic status [27], and the improvement of learning frequency [28] are all important mediating mechanisms.

Second, Internet use can have a negative association with health. Matusitz et al. found that Internet use has a negative association with the health of Americans. The reason is that Internet use may encourage prolonged sitting, promote inactivity, and increase the risk of obesity [29]. Hökby et al. found that the time spent on the Internet and different ways of using the Internet have a negative association with the health of adolescents [30]. In a study on 1248 adolescents in South Korea, Choi et al. found that excessive Internet use may have a bad association with the health of adolescents [31]. Ning et al. found that excessive Internet use can have a negative connection with the health of rural youth based on the data from the China Family Nutrition and Health Survey [32]. Third, there is a curvilinear relationship between Internet use and health; that is, when Internet use exceeds a certain level, health will diminish. For example, Bélanger et al. [33] and Lu and Wang confirmed that excessive Internet use may be harmful to health [34].

In addition, some authors have discussed the relationship between the Internet use and health behavior of the population. Webb et al. found that the Internet is increasingly used as a medium for the delivery of interventions designed to control health behavior. However, recent reviews of these interventions have not systematically identified the characteristics of such interventions not the effectiveness associated with them to date [35]. Whittaker et al. investigated whether mobile phone application-based smoking cessation interventions increase the rate of smoking cessation in smokers who smoke and who want to quit and found beneficial effects of these smoking cessation interventions on six-month cessation outcomes [36]. Afshin et al. evaluated 244 studies on the association with Internet and mobile phone use on healthy lifestyles and revealed that the Internet can intervene important lifestyle behaviors for up to 1 year [37].

The literature review revealed that several studies have confirmed that Internet use is associated with health. However, a consensus of the conclusion has not been reached, and the mechanism analysis focuses on the search for health-related information. The study of health behavior as a mediating mechanism has not yet attracted the attention of academic circles. The research on Internet use and health behavior has mostly put forward policy recommendations from a qualitative perspective, and there is a lack of relevant quantitative analyses.

## 3. Data and Methods

### 3.1. Data Sources

In this study, data were obtained from the CFPS in 2018. The data collection began in 2010 and was implemented by the Chinese Social Science Survey Center of Peking University. The survey covered 25 provinces (municipalities and autonomous regions) across the country. It had a large sample size and strong representativeness. The questionnaire was developed by the Social Science Survey Center of Peking University. The survey involved 16,000 households, and it was conducted once every 2 years. The Chinese Social Science Survey Center of Peking University took on the questionnaire management and the updating of the scientific, open access database. The 2018 data included family member questionnaires, adult self-report questionnaires, and children’s questionnaires. This study focused on Internet use and personal health; therefore, the adult questionnaire was selected as the research sample. There were 37,354 samples in the adult data. After processing and eliminating the missing values, outliers, and invalid variables, 8306 valid samples were finally obtained.

### 3.2. Variable Design

#### 3.2.1. Dependent Variables

The health level of the population was the dependent variable. In previous studies, the health measurement standards were different. Some studies have used physical and mental health to measure health [38,39], whereas some have used self-rated health to represent the health of the population [40,41]. The validity and credibility of this indicator have been confirmed by most studies. The present study used self-rated health and chronic conditions to measure the health level of the population. Self-rated health was measured by the question (Question P201) “Could you please comment on your own health?” The answer had five categorical variables, in which 1 represented unhealthy, and 5 represented very healthy: the higher the score, the better the health. Chronic conditions were measured and answered by the question (P401) “Do you have a chronic disease?” The answer was a binary variable, where 0 indicated unhealthy, and 1 healthy.

#### 3.2.2. Independent Variables

The core independent variable of this study was Internet usage. The questionnaire included questions such as the following: “Do you use a mobile device (such as a mobile phone and tablet) to go online? Do you use a computer to go online?” This study combined the two questions. If the answers were both yes, the sample was considered as using the internet, and the question was scored as 1; otherwise, it was scored as 0. In addition, to ensure the robustness of the model’s estimation results, “using the Internet for social frequency” was selected as a substitute variable for analysis, with very frequent (1), more frequent (2), less (3), and never (4) representing the choices, and the frequency decreased as the value increased.

#### 3.2.3. Mediating Variables

The health behavior of the population was the intermediary variable. Previous studies [42,43,44] have revealed that smoking, drinking, TV time, and exercise duration are all important indicators of healthy behaviors. Therefore, smoking status, frequency of drinking, TV time, and exercise duration were selected to comprehensively measure healthy behaviors. According to the World Health Organization’s criteria for judging health behaviors, if a person smokes, drinks frequently, watches more than 15 h of TV per week, and exercises less than 150 min per week, he/she is considered to have poor health behaviors and is given a score of 0; otherwise, a score of 1 is given.

#### 3.2.4. Control Variables

With reference to previous studies, the present study incorporated individual characteristic variables, such as sex, age, marital status, education level, political status, medical insurance, and other variables, into the model [34,45]. In addition, considering that the health level of the population is mostly affected by lifestyle, this study incorporated smoking, drinking, and staying up late into the model to ensure the accuracy of the model’s estimated results. According to the descriptive results, on an average, the respondents were 32.351 years old. Regarding Internet use, the proportion of the population using the Internet was 41.45%, which was still far from the proportion of China’s Internet penetration rate (70.4%). The proportion of men in the sample population was 52.09%, which was slightly higher than the amount of women. In terms of marriage, the highest proportions of married and unmarried people were 75% and 21.29%, respectively. In addition, 56.27% of the population had a high school level of education and above, 25.75% had college education, and 17.41% had elementary school education and below. The descriptive statistics of the variables are shown in Table 1. In addition, we performed a collinearity test and the results are shown in Appendix A Table A1.

### 3.3. Statistical Analysis

In this study, the explanatory variables were five- and two-category variables. Therefore, different measurement models were set up for analysis. The ordered Probit regression model [46,47] was set for five categorical variables:

(1) $${\mathrm{Health}}_{i}=\alpha +{\beta \mathrm{Internet}}_{i}+{\gamma Z}_{i}+\varepsilon_{i}$$

In Equation (1), ${\mathrm{Health}}_{i}$ represents the health level of a person in China. ${\mathrm{Internet}}_{i}$ represents Internet use, $Z_{i}$ is the control variable that affects the health status of the population, α is the intercept term, β and γ represent the regression coefficients for the corresponding variables, and $\varepsilon_{i}$ represents the error term. A Probit regression model was set up for the binary variables as follows:

(2) $$\mathrm{Pr}(Y_{i}=1)=\phi (\alpha +{\beta \mathrm{Internet}}_{i}+{\gamma Z}_{i}+\varepsilon_{i})$$

In Equation (2), i represents a resident, $Y_{i}$ represents the health of a resident i, ${\mathrm{Internet}}_{i}$ represents Internet use, $Z_{i}$ represents the control variable included in the model, $\varepsilon_{i}$ represents the error term, and β and γ represent the regression coefficients for the corresponding variables.

Internet use is affected by several factors, such as the individual’s age, occupation type, and education level, and is an independent choice made by the individual. Therefore, the model results may be affected by sample selection bias. To control the sample selection bias as much as possible in order to the model estimation, the study used the PSM model [48] to estimate the net effect of Internet use on the health of the population.

**Figure 1:**
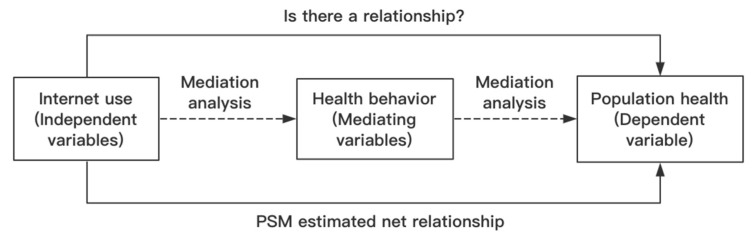
Hypothesis between the research relationships of variables.

Propensity score matching (PSM) is a statistical method that can be used to process observational study data [49]. For various reasons, observational research may have more data biases and confounding variables; thus, PSM is used to reduce their effects [50]. This method was first proposed by Paul Rosenbaum and Donald Rubin in 1983 and is commonly used in medicine, public health, economics, and other fields [51]. The main purpose of PSM is to match similar samples by covariates to obtain pure estimation results. All of the analyses were conducted using STATA (version 15.0, StataCorp., College Station, TX, USA). The methods corresponding to individual content are presented in Table 2. The hypothesis of the relationship between the variables is shown in Figure 1.

## 4. Results

### 4.1. Basic Regression

Depending on the type of variable, different econometric models were used for estimation in this study, and the estimation results are shown in Table 3. We found that internet use has a significant association with both the self-rated health and chronic conditions of the population. The results from Model (1) revealed that when no control variables were included, the self-rated health of Internet users was higher by 0.058 Probit units in the direction of good health compared to that of non-users. The results of Model (3) revealed that when no control variables were excluded, the self-rated health of Internet users was higher by 0.088 probit units in the direction of good health than that of non-users. However, with the inclusion of control variables, the results were significant at a 1% level of significance, with a 4.0% probability that the chronic conditions of Internet users changed in a better direction than that of non-users. The 95% confidence interval results are shown in Appendix B Table A2.

Because subjective self-rated health is a five-category variable in this study, the data in Table 3 reflect the extent to which Internet use affects the health of the population rather than the marginal effects. Therefore, we further examined the marginal effects of Internet use on the subjective health of the population by combining the cut-point values, and the results are presented in Table 4. As shown in Table 4, compared to the health of non-users, the probability of the self-rated health of Internet users being “general” and “unhealthy” both decreased by 0.9%, whereas the probability of being “very healthy”, “relatively healthy”, and “healthier” increased by 18.8%, 1.0%, and 10.9%, respectively.

The estimates of the control variables were generally in line with expectations. In terms of individual characteristics, men have better self-rated health than women, whereas women have better chronic conditions than men. Regarding age, as individuals mature, their body functions continue to decline, and their health deteriorates. Compared to the less-educated population, the more-educated population was healthier. Political status had a significant positive association with the self-rated health of the population at a 1% level. Regarding work attributes, compared to non-agricultural workers, agricultural workers had poorer health. From the perspective of lifestyle, the frequencies of physical exercise, smoking, drinking, and staying up late were associated with health quality. The higher the frequency of physical exercise, the better the self-rated health of the population.

### 4.2. Robustness Test

The robustness test refers to the examination of the robustness of the evaluation methods and index interpretation capabilities; that is, when certain parameters are changed, the evaluation methods and indicators are assessed to check whether they still maintain a relatively consistent and stable interpretation of the evaluation results [52]. We used both substitution measures and the substitution of the core explanatory variables to conduct the robustness tests of the model. As the explanatory variables were five- and two-category variables, the ologit and logit models were used as replacement measures for the estimation of the results. Regarding the Internet usage variables of the population, the 2018 CFPS data also included the question “How often do you use the Internet for social interaction?” We used the rating of the population who used the Internet for social interaction as the core explanatory variable to replace Internet use for robustness testing. As presented in Table 5, Models (1) and (2) provide estimates after replacing the measures. The results revealed that Internet use demonstrated a significantly positive association with the self-rated health and chronic conditions of the population at a 1% level, which is consistent with the results of the baseline regression. Models (3) and (4) provided estimates after replacing the core explanatory variables. As the frequency of using the Internet for social interaction increases, the health link for the population increases significantly, which is also consistent with the baseline regression results. These results suggest that the estimation results of the model in this study have good robustness. The 95% confidence interval results are shown in Appendix C Table A3.

### 4.3. PSM to Eliminate Sample Selection Bias

To overcome the problem of sample self-selection bias, we used a PSM model to estimate the net association of Internet use on the self-rated health and the chronic conditions of the population. In this study, three methods, namely K-nearest neighbour matching, radius neighbour matching, and kernel matching, were used for estimation. To ensure a good match, a balanced test of the quality of the sample pie was required on top of the propensity score estimated using the logit model. If the difference between the two groups of samples after matching was significant, the matching was considered poor, and the estimation was invalid; however, if the difference was non-significant, then the matching was considered better [53]. The results of the balance test are shown in Table 6.

According to the data presented in Table 6, the absolute value of the standardized deviation after matching for all variables is <5%. From the results of the *t*-test of means, the variables in the treatment and control groups had significant t-values before matching and were not significant after matching, with the exception of the individual variables. Therefore, no systematic differences were found between the matched treatment and control groups, effectively addressing the problem of sample selection bias. The average treatment effects of Internet use on the self-rated health and chronic conditions of the population are reported in Table 7.

According to the data exhibited in Table 7, the average treatment effects on the self-rated health and chronic conditions of the population before matching were 0.056 and 0.004, respectively, and the results after matching were 0.103 and 0.023, respectively, using the K-nearest neighbour matching method. After controlling for sample selection bias, the net association of Internet use on the self-rated health and chronic conditions of the population were 10.3% and 2.3%, respectively. Radius neighbour matching and kernel matching yielded results that were similar to those of K-nearest neighbour matching. The net association of Internet use on the self-rated health of the population was 9.7% and 8.5%, and the net association on chronic conditions was 2.2% and 2.3% by radius neighbour matching and kernel matching, respectively. The results obtained by PSM were robust and suggested that without the elimination of the sample selection bias, the relationship between Internet use and the health of the population would be underestimated.

### 4.4. Regression Results in Different Subgroups

Disparities exist in economic levels and in the infrastructure among regions of China, such as between urban and rural areas, between types of occupation, and between levels of education. As numerous studies have analysed this from the perspective of sex and age, this study further examined the heterogeneous association of Internet use on the health of the population with respect to types of occupation (agriculture versus non-agriculture sector) and literacy. As reflected in Table 8, Internet use has a more significant association with the health of non-agricultural workers and those with higher levels of education than that of agricultural workers and those with lower levels of education.

### 4.5. Mediation Analysis

**Figure 2:**
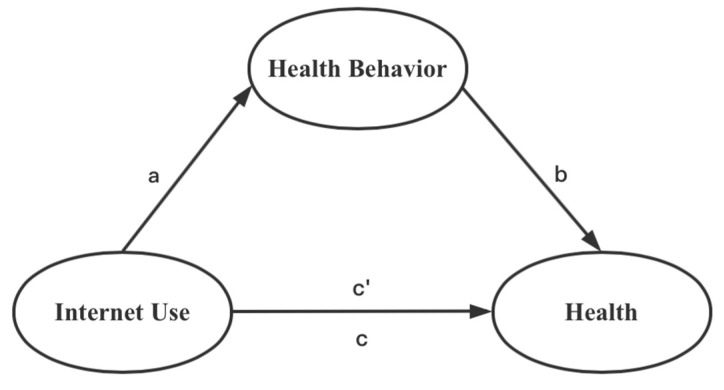
Mediation path of health behavior.

Existing studies on the mechanisms underlying the association between Internet use and health are unclear. Health behavior is the foundation of population health. Therefore, for further exploration, we selected health behavior as the mediating variable to verify its mediating mechanism. Based on previous research, this study referred to the basic ideas of the analysis by Baron and Kenny [54] and adopted a stepwise regression method to verify the mediation. We also calculated the path coefficients. Figure 2 shows the mediation pathway. Table 9 provides the estimated results.

According to the calculation results, Internet use had a significant association with both subjective self-rated health (*c* = 0.078, *p* < 0.01) and chronic conditions (*c* = 0.040, *p* < 0.01). Moreover, Internet use had a significantly positive association with health behavior (*a* = 0.139, *p* < 0.05). When both Internet use and health behavior were included in the model, this finding remained. Coefficient b (0.115) and coefficient c’ (0.075) in the regression results of Model (2) are both significantly positive at the 1% significance level. These results indicated a significant mediation between health behavior and the association of Internet use with health. According to the calculations, the mediation of self-rated health was 0.0159 (a * b), which accounted for 20.38%. Similarly, the mediation of chronic conditions was 0.0118 or 29.53%. Table 10 shows the results of the mediation coefficient path.

## 5. Discussion

### 5.1. Summary of the Finding

In this study, we used data from the 2018 CFPS to examine the relationship between internet use and the health of the population by assessing the possible heterogeneity of this association across groups and mediating mechanisms. The results revealed that Internet use has a significant positive association with population health, which is consistent with the findings of some existing studies, such as those by Bessière, Yang and He, Zhu et al., and Neter et al. [22,25,55,56] Moreover, Internet use had a more positive association with self-rated health than it did with chronic conditions. However, the size of adj-R^2^ in Models 1 and 3 (Table 3 and Table 5) indicates that the association between Internet use and population health are small and of limited interest. This may be somewhat related to the fact that Models 1 and 3 did not include control variables in the model. The results of the heterogeneity analysis revealed that the relationship between Internet use and health was different among occupational groups; that is, Internet use demonstrated a more significant association with the health of non-agricultural workers than that of agricultural workers. The possible reason is that the vast majority of agricultural workers live in rural areas. China is a large agricultural country with a large population of farmers. A gap remains between the level of infrastructure development and public services in rural areas compared to in urban areas in China, and this gap may lead to a lower Internet penetration rate in rural areas [57]. In terms of education, There are significant differences between the effects that internet use has on the health of people with different education levels. The Internet has a positive association with the health of people with high education levels compared to those with low education levels, and this finding is consistent with the results of the study by Li and Peng [58,59]. This is because more educated people have less difficulty using the Internet, are good at using Internet tools to obtain health knowledge, and have a better level of health knowledge, whereas less educated people may find it difficult to use the Internet.

The results of the mediation analysis revealed that Internet use has a positive association with the health behaviors of the population, which is consistent with the findings of previous studies [36,37]. The Internet can broaden the population’s access to health knowledge and break the original medium of knowledge dissemination; as a result, the population can easily obtain health knowledge through various new media channels; this, it has a positive association with health behavior. The Internet, as a tool for media communication, can be also valid for knowledge dissemination. That is, people can learn about health while browsing health-related websites.

### 5.2. Policy Implication

The conclusions reached in this study have several important policy implications. First, the spread of the Internet in rural areas should be further accelerated. According to relevant data, China has nearly one billion Internet users; however, the Internet penetration rate in rural areas is only 55.9%. Given the dualistic nature of China’s urban–rural distribution, the economic development and information infrastructures in rural areas lag far behind those in urban areas [17]. Therefore, accelerating Internet access in rural areas is now a top priority for the Chinese government. The pace of information infrastructure construction in rural areas should be accelerated, and infrastructure construction should be steadily promoted in phases and by target groups in response to the variations between different rural areas. Concomitantly, the government should increase education on the use of the Internet in rural areas and should adopt a hands-on approach to advocate for less-educated farmers regarding the use of the Internet so that the rural Chinese population can benefit from the Internet. Second, multiple Internet channels should be used to popularize the health knowledge of the population. As an effective source of health-related information, the Internet is associated with changes in people’s attitudes and behaviors, thus contributing to disease prevention and improving the quality of life and health standards of the population. With the high popularity of short videos such as TikTok videos, health science videos can be produced to educate the population on health through easy-to-understand forms and content, thus enhancing their health literacy.

### 5.3. Strengths

This study has several strengths. First, this study validated the mediating role of health behavior. It not only complemented previous studies and broadened the horizons of the academic field, but it also provided new evidence for understanding the health of the population. Second, we used the latest CFPS data, which better reflected the new state of Internet usage among the Chinese population and its association with their health. Third, this study used PSM models to estimate the net association of Internet use on health, eliminating endogeneity problems due to sample selection bias and ensuring the robustness of the model estimates. Fourth, this paper used both self-rated health and chronic conditions measures to provide a more comprehensive representation of the health of the population, reducing errors to some extent.

### 5.4. Limitations

Several limitations should be noted. First, due to the limited data resources, the relationship between Internet use and population health needs to be further explored and explained in future studies. Second, the only independent variable was Internet use, and there are different ways to use the internet as well as multiple types of content online (e.g., using the Internet to search for health information, using the Internet for entertainment, etc.). Therefore, we will further study the effect of different content and methods of Internet use on the health of the population. Third, this article only studies the relationship between Internet use and the subjective health of the population and lacks a description of objective health. In the future, we will collect clinical data to further study the relationship between Internet use and the objective health of the population. It should also be noted that the size of adj-R^2^ in Models 1 and 3 (Table 3 and Table 5) indicates that the association between Internet use and population health are small and of limited interest. Therefore, in future studies, we will select a larger sample and more objective variables to further validate the relationship between Internet use and population health.

## 6. Conclusions

This study focused on the association of Internet use on subjective health and assessed the possible heterogeneity of this association among groups and its mediation path. The results revealed that Internet use has a significant positive association with population health. This finding is still valid after the use of PSM to eliminate sample selection bias. In addition, there are certain differences between the relationships of Internet use on the self-rated health and the chronic conditions of the population. Internet use has a greater association with self-rated health than it does on chronic conditions. Moreover, the association of Internet use on health shows significant heterogeneity in terms of occupational type and educational level. Compared to agricultural workers and those with lower education levels, Internet use has a more significant association with the health of non-agricultural workers and those with higher education levels. Finally, the mediation analysis revealed that health behavior is an important channel connecting the relationship between Internet use and population health, with a partial mediation between the two.

## Figures and Tables

**Table 1:** Descriptive statistics of variables.

Variables	Definition	N	Percentage (%)
Dependent variable			
Self-rated health	Unhealthy = 1	502	6.04
	General = 2	746	9.20
	Healthier = 3	3942	47.46
	Relatively healthy = 4	1729	20.82
	Very healthy = 5	1369	16.48
Chronic conditions	Unhealthy = 0	1988	23.93
	Healthy = 1	6318	76.07
Independent variable			
Internet use	No = 0	4863	58.55
	Yes = 1	3443	41.45
Internet social frequency	Very frequently = 1	6836	82.30
	Frequently = 2	878	10.57
	Less = 3	142	1.71
	Never = 4	450	5.42
Personal characteristics			
Sex	Female = 0	3979	47.91
	Male = 1	4327	52.09
Age	Unit: Years	8306	-
Marital status	Unmarried = 1	1768	21.29
	Married = 2	6250	75.25
	Cohabitation = 3	41	0.49
	Divorced = 4	217	2.61
	Widowed = 5	30	0.36
Education	Primary school and below = 1	1446	17.41
	Middle school = 2	4674	56.27
	College = 3	2139	25.75
	Postgraduate = 4	47	0.57
Political status	Non-party members = 0	8109	97.63
	Party member = 1	197	2.37
Domicile	Agricultural = 1	6177	74.37
	Non-agricultural = 2	2127	25.61
Work attributes	Agricultural work = 1	1431	17.23
	Non-agricultural work = 2	6875	82.77
Medical insurance	No = 0	851	10.25
	Yes = 1	7455	89.75
Lifestyle			
Smoking	No = 0	5640	67.90
	Yes = 1	2666	32.10
Drinking	No = 0	7153	86.12
	Yes = 1	1153	13.88
Staying up late	No = 0	6931	83.45
	Yes = 1	1375	16.55
Mediating variables			
Health behavior	Poor= 0	3757	45.23
	Good = 1	4549	54.77

Notes: (1) In China, primary school, secondary school, and college span 6 years, 6 years (including middle school and high school), and 3–4 years, respectively. Postgraduate education requires 3–4 years (including master’s and doctoral degrees). (2) Political status refers to whether the individual is a member of the party.

**Table 2:** Methods corresponding to different contents.

Contents	Methods
Basic regression	Order probit/Probit
Robustness test	Ologit/Order probit/Probit
Net relationship between Internet use and health	Propensity Score Matching (PSM)
Regression results in different subgroups	Order probit/probit
Mediation analysis	Stepwise regression

**Table 3:** Regression of Internet use on population health benchmarks.

Variable	Model (1)	Model (2)	Model (3)	Model (4)
	Self-Rated Health	Self-Rated Health	Chronic Conditions	Chronic Conditions
Independent variable				
Internet use	0.058 ** (0.024)	0.078 *** (0.028)	0.088 ** (0.042)	0.040 *** (0.050)
Personal characteristics				
Sex		0.129 *** (0.031)		−0.008 * (0.055)
Age		−0.030 *** (0.002)		−0.027 *** (0.003)
Marriage		0.002 (0.023)		0.004 (0.040)
Education		0.040 * (0.022)		0.022 (0.038)
Political status		0.245 *** (0.078)		−0.013 (0.140)
Domicile		−0.027 (0.030)		−0.015 * (0.009)
Work attributes		0.064 * (0.034)		0.170 *** (0.056)
Medical insurance		−0.040 (0.039)		−0.083 (0.076)
Lifestyle				
Exercise frequency		0.017 *** (0.004)		−0.010 (0.007)
Smoking		0.054 * (0.033)		0.107 * (0.059)
Drinking		0.079 ** (0.036)		0.080 (0.065)
Staying up late		−0.188 *** (0.033)		−0.128 *** (0.058)
Observations	8306	8306	8306	8306
Adj-R^2^	0.0003	0.0214	0.0010	0.0333

Note: Standard errors in brackets, * *p* < 0.1, ** *p* < 0.05, *** *p* < 0.01.

**Table 4:** Marginal effects of Internet use on subjective self-evaluation of health.

Variable	Y = 1	Y = 2	Y = 3	Y = 4	Y = 5
Self-rated health	Very healthy	Relatively healthy	Healthier	General	Unhealthy
Internet use	0.188 *** (0.006)	0.010 *** (0.003)	0.109 *** (0.004)	−0.009 *** (0.003)	−0.009 *** (0.003)

Note: Standard errors in brackets, *** *p* < 0.01.

**Table 5:** Robustness test results.

Variable	Model (1)	Model (2)	Model (3)	Model (4)
	Self-Rated Health	Chronic Conditions	Self-Rated Health	Chronic Conditions
Independent variable				
Internet use	0.127 *** (0.048)	0.092 *** (0.103)		
Frequency of using the Internet socially			−0.039 *** (0.031)	−0.028 *** (0.026)
Personal characteristics				
Sex	0.237 *** (0.054)	−0.012 (0.110)	0.121 *** (0.031)	−0.009 (0.054)
Age	−0.055 *** (0.003)	−0.051 *** (0.006)	−0.030 *** (0.002)	−0.026 *** (0.003)
Marriage	0.012 (0.039)	−0.004 (0.081)	0.005 (0.023)	0.004 (0.040)
Education	0.071 * (0.039)	0.050 (0.078)	0.020 (0.021)	0.007 (0.035)
Political status	0.416 *** (0.134)	−0.002 (0.287)	0.240 *** (0.078)	−0.014 (0.140)
Domicile	−0.047 (0.051)	−0.026 * (0.015)	−0.035 (0.030)	−0.016 * (0.009)
Work attributes	0.095 (0.061)	0.334 *** (0.110)	0.059 * (0.034)	0.159 *** (0.056)
Medical insurance	−0.039 (0.068)	−0.188 (0.159)	−0.040 (0.034)	−0.086 (0.076)
Lifestyle				
Exercise frequency	0.031 *** (0.008)	−0.020 (0.014)	0.017 *** (0.004)	−0.010 (0.007)
Smoking	0.098 * (0.057)	0.217 * (0.119)	0.056 * (0.033)	0.109 * (0.059)
Drinking	0.144 *** (0.064)	0.165 (0.135)	0.080 ** (0.036)	0.078 (0.065)
Staying up late	−0.323 *** (0.057)	−0.239 ** (0.118)	−0.192 *** (0.033)	−0.133 ** (0.058)
Observations	8306	8306	8306	8306
Adj-R^2^	0.0222	0.0325	0.0211	0.0334

Note: Standard errors in brackets, * *p* < 0.1, ** *p* < 0.05, *** *p* < 0.01.

**Table 6:** Quality balance test for sample matching.

Variable	Unmatched Matched	Mean		Bias (%)	Reduce Bias (%)	*t*-Test	
		Treated	Control			*t*	*p* > |*t*|
Sex	U	0.565	0.490	15.0	74.3	6.72	0.000
	M	0.564	0.545	3.9		1.60	0.109
Age	U	30.394	33.737	−44.7	90.8	−19.72	0.000
	M	30.403	30.097	4.1		1.89	0.059
Marriage	U	1.740	1.937	−34.3	93.0	−15.46	0.000
	M	1.742	1.756	−2.4		−1.00	0.318
Education	U	2.470	1.829	108.8	99.2	48.98	0.000
	M	2.466	2.461	0.9		0.37	0.709
Political status	U	0.038	0.136	15.5	99.2	7.24	0.000
	M	0.038	0.037	0.1		0.04	0.966
Domicile	U	1.386	1.166	50.4	92.9	23.17	0.000
	M	1.383	1.368	3.6		1.34	0.182
Work attributes	U	1.944	1.746	56.8	98.1	24.17	0.000
	M	1.944	1.940	1.1		0.66	0.507
Medical insurance	U	0.892	0.902	−3.1	77.2	−1.41	0.158
	M	0.892	0.890	0.7		0.29	0.773
Exercise frequency	U	2.167	1.732	16.5	97.6	7.38	0.000
	M	2.160	2.149	0.4		0.16	0.870
Smoking	U	0.313	0.327	−3.0	31.4	−1.34	0.180
	M	0.314	0.323	−2.0		−0.85	0.395
Drinking	U	0.130	0.145	−4.6	97.5	−2.06	0.040
	M	0.130	0.130	0.1		0.05	0.961
Staying up late	U	0.217	0.129	23.5	87.4	10.74	0.000
	M	0.216	0.205	3.0		1.13	0.257

**Table 7:** Propensity score matching estimation results.

	Self-Rated Health				Chronic Conditions			
	Treated	Control	ATT	SE	Treated	Control	ATT	SE
Unmatched	2.642	2.698	0.056	0.023	0.237	0.241	0.004	0.010
Matched								
(1)	2.645	2.252	0.103	0.037	0.237	0.215	0.023	0.015
(2)	2.646	2.550	0.097	0.034	0.237	0.216	0.022	0.014
(3)	2.645	2.560	0.085	0.033	0.237	0.214	0.023	0.013

Note: (1) K-nearest neighbour matching, (2) radius neighbour matching and (3) kernel matching. K-nearest neighbour matching used “one-to-four” matching, radius neighbour matching had a radius of 0.01, and kernel matching used default values for both kernel function and bandwidth.

**Table 8:** Analysis of work attributes and educational heterogeneity.

Variable	Work Attributes				Education			
	Agricultural		Non-Agricultural		High School and Below		University and Above	
	(1)	(2)	(1)	(2)	(1)	(2)	(1)	(2)
Internet use	0.012 (0.087)	0.052 (0.156)	0.086 *** (0.029)	0.055 ** (0.054)	0.043 (0.032)	0.015 (0.058)	0.103 * (0.058)	0.039 * (0.108)
Control variables	Yes	Yes	Yes	Yes	Yes	Yes	Yes	Yes
Observations	1431	1431	6875	6875	6120	6120	2186	2186
Adj-R^2^	0.0219	0.0444	0.0206	0.0275	0.0216	0.0378	0.0249	0.0516

Note: Standard errors in brackets, * *p* < 0.1, ** *p* < 0.05, *** *p* < 0.01; (1) self-rated health and (2) chronic conditions.

**Table 9:** Mediation of health behavior.

	Self-Rated Health			Chronic Conditions		
Variables	Step One	Step Two	Step Three	Step One	Step Two	Step Three
Internet use	0.078 *** (0.028)	0.139 *** (0.046)	0.075 *** (0.276)	0.040 *** (0.050)	0.139 *** (0.046)	0.038 *** (0.051)
Control variables	Yes	Yes	Yes	Yes	Yes	Yes
Health behavior			0.115 *** (0.120)			0.085 *** (0.094)
Observations	8306	8306	8306	8306	8306	8306
Adj-R^2^	0.0214	0.0541	0.0255	0.0333	0.0541	0.0334

Note: standard errors in brackets, *** *p* < 0.01.

**Table 10:** Result of mediation path coefficient.

Variables	c	a	b	c’	Mediation	Percentage (%)
Self-rated health	0.078	0.139	0.115	0.075	0.0159	20.38
Chronic conditions	0.040	0.139	0.085	0.038	0.0118	29.53

Note: The result of the mediation is a * b; percentage indicates the proportion of mediation in the total.

## Data Availability

The data of CFPS2018 is publicly available at http://www.isss.pku.edu.cn/cfps/ accessed on 13 January 2021.

## Appendix A

**Table A1:** Results of collinearity test.

Variable	VIF
Sex	1.73
Age	1.69
Marriage	1.42
Education	1.41
Political status	1.25
Domicile	1.24
Work attributes	1.20
Medical insurance	1.14
Exercise frequency	1.07
Smoking	1.03
Drinking	1.02
Staying up late	1.02

## Appendix B

**Table A2:** The 95% confidence interval of the basic regression.

Variable	Subjective Health		Subjective Recent Health	
	95% Confidence Interval		95% Confidence Interval	
Internet use	0.011	0.104	−0.046	0.075
Sex	0.068	0.190	0.105	0.263
Age	−0.034	−0.027	−0.016	−0.007
Marriage	−0.043	0.046	−0.043	0.072
Education	−0.003	0.084	0.022	0.135
Political status	0.092	0.399	−0.196	0.202
Domicile	−0.086	0.031	−0.059	0.093
Work attributes	−0.003	0.131	0.029	0.199
Medical insurance	−0.117	0.037	−0.102	0.098
Exercise frequency	0.009	0.026	−0.012	0.011
Smoking	−0.010	0.118	−0.114	0.055
Drinking	0.008	0.151	−0.001	0.187
Staying up late	−0.252	−0.124	−0.292	−0.130

## Appendix C

**Table A3:** The 95% confidence interval of the robustness test.

Variable	Model (1)		Model (2)		Model (3)		Model (4)	
	95% Confidence Interval				95% Confidence Interval			
Internet use	0.015	0.175	−0.078	0.127	−0.070	−0.010	−0.052	0.026
Sex	0.132	0.342	0.181	0.454	0.061	0.182	0.096	0.254
Age	−0.061	−0.048	−0.027	−0.012	−0.034	−0.026	−0.016	−0.007
Marriage	−0.065	0.089	−0.075	0.123	−0.039	0.050	−0.038	0.077
Education	−0.005	0.147	0.039	0.233	−0.022	0.061	0.001	0.107
Political status	0.153	0.678	−0.331	0.358	0.087	0.394	−0.201	0.197
Domicile	−0.148	0.054	−0.100	0.159	−0.094	0.023	−0.069	0.082
Work attributes	−0.024	0.213	0.053	0.339	−0.008	0.126	0.023	0.193
Medical insurance	−0.173	0.095	−0.175	0.168	−0.117	0.038	−0.102	0.099
Exercise frequency	0.015	0.046	−0.020	0.019	0.008	0.025	−0.013	0.010
Smoking	−0.013	0.210	−0.198	0.094	−0.008	0.121	−0.111	0.057
Drinking	0.019	0.269	−0.001	0.325	0.009	0.151	0.001	0.188
Staying up late	−0.434	−0.212	−0.495	−0.221	−0.256	−0.128	−0.297	−0.135
